# Early Experiences With COVID-19 Testing in Transplantation

**DOI:** 10.1097/TXD.0000000000001024

**Published:** 2020-06-11

**Authors:** Brian J. Boyarsky, Allan B. Massie, Arthur D. Love, William A. Werbel, Christine M. Durand, Robin K. Avery, Kyle R. Jackson, Amber B. Kernodle, Alvin G. Thomas, Matthew Ronin, Michelle Altrich, Patricia Niles, Chad Trahan, Jonathan Hewlett, Dorry L. Segev, Jacqueline M. Garonzik-Wang

**Affiliations:** 1 Department of Surgery, Johns Hopkins University School of Medicine, Baltimore, MD.; 2 Department of Epidemiology, Johns Hopkins School of Public Health, Baltimore, MD.; 3 Department of Medicine, Johns Hopkins University School of Medicine, Baltimore, MD.; 4 National Kidney Registry, Babylon, NY.; 5 Viracor Eurofins, Lee’s Summit, MO.; 6 Southwest Transplant Alliance, Dallas, TX.

## Abstract

**Background.:**

The early effects of coronavirus disease 2019 (COVID-19) on transplantation are dramatic: >75% of kidney and liver programs are either suspended or operating under major restrictions. To resume transplantation, it is important to understand the prevalence of COVID-19 among transplant recipients, donors, and healthcare workers (HCWs) and its associated mortality.

**Methods.:**

To investigate this, we studied severe acute respiratory syndrome coronavirus 2 diagnostic test results among patients with end-stage renal disease or kidney transplants from the Johns Hopkins Health System (n = 235), and screening test results from deceased donors from the Southwest Transplant Alliance Organ Procurement Organization (n = 27), and donors, candidates, and HCWs from the National Kidney Registry and Viracor-Eurofins (n = 253) between February 23 and April 15, 2020.

**Results.:**

We found low rates of COVID-19 among donors and HCWs (0%–1%) who were screened, higher rates of diagnostic tests among patients with end-stage renal disease or kidney transplant (17%–20%), and considerable mortality (7%–13%) among those who tested positive.

**Conclusions.:**

These findings suggest the threat of COVID-19 for the transplant population is significant and ongoing data collection and reporting is critical to inform transplant practices during and after the pandemic.

Coronavirus disease 2019 (COVID-19) has rapidly impacted the world.^1-5^ Information about its impact on transplant candidates, recipients, donors, and healthcare workers (HCWs) has been limited to case series, shared knowledge, and expert discussion.^6-11^ The early effects of COVID-19 on the field are remarkable: in a national survey of transplant centers in the United States, >75% of kidney and liver programs were either suspended or operating under major restrictions, which has significant implications for those awaiting transplantation.^12^ To resume transplantation, it is important to understand the prevalence of COVID-19 and its associated mortality.

Given testing shortages and lack of national infrastructure, there is a dearth of information in the United States on COVID-19 prevalence among the general population. In a screening study from Iceland, severe acute respiratory syndrome coronavirus 2 (SARS-CoV-2) was detected in 0.6%–0.8% of the population.^13^ Notably, among those who tested positive, 43% were asymptomatic. As the donor pool is often reflective of the general population, these data are critical, as donor-derived SARS-CoV-2 transmission could theoretically have devastating effects on a newly transplanted patient. Among those infected with SARS-CoV-2, mortality has ranged from 0.06% to 13.4%,^14–19^ highest among the elderly, those with comorbidities, and the immunocompromised.^20–22^ The uncertainty about disease prevalence and morbidity/mortality have led to a significant reduction in transplantation, and to resume practice, we need data to help inform practice.

We used data from Johns Hopkins Health System (JHHS), the Southwest Transplant Alliance (STA) organ procurement organization, and National Kidney Registry (NKR) to quantify SARS-CoV-2 in transplant candidates, recipients, donors, and HCWs. Our findings can help inform transplant practice during these uncertain times.

## MATERIALS AND METHODS

### Johns Hopkins Health System

#### Data Source

We conducted a retrospective review of electronic medical records from the JHHS using the Epic SlicerDicer feature between March 1 and April 15, 2020. Headquartered in Baltimore, MD, JHHS unites physicians of the Johns Hopkins University School of Medicine with the organizations, health professionals, and facilities of The Johns Hopkins Hospital and Health System. Johns Hopkins has 6 academic and community hospitals, 4 suburban healthcare and surgery centers, >40 patient care locations, and a home care group. Annually, JHHS receives nearly 3 million patients, 360,000 emergency room visits, and 900,000 outpatient visits. We included records of patients with end-stage renal disease (ESRD) or history of kidney transplant (KT) who underwent diagnostic SARS-CoV-2 testing. We recorded the outcome of the test, as well as demographic characteristics (age, sex, race) and vital status. To define a history of ESRD, we used the International Statistical Classification of Diseases-10 code N18.6. We included ESRD patients who were dialysis-dependent. To define a history of kidney transplant, we used the International Statistical Classification of Diseases-10 code Z94.0. To define a SARS-CoV-2 test, we used any of the following: nasopharyngeal swab, sputum, or bronchoalveolar lavage. We then recorded characteristics of patients who tested positive. This study was approved by the Johns Hopkins Medical Institutions Institutional Review Board (IRB00247940).

### Southwest Transplant Alliance Organ Procurement Organization

#### Data Source

We reviewed deceased donor (DD) screening data from the STA organ procurement organization between February 23 and April 13, 2020. STA is affiliated with 10 transplant centers and 270 hospitals in 89 Texas counties. We recorded demographic characteristics of donors (age, sex, race), clinical characteristics (cause of death, SARS-CoV-2 polymerase chain reaction (PCR) result), and transplant characteristics (donation after brain death versus donation after circulatory death, organs transplanted).

### NKR and Viracor-Eurofins

#### Data Source

We reviewed donor and recipient screening data from NKR and Viracor-Eurofins. Both companies use the SARS-CoV-2 reverse transcriptase-PCR (RT-PCR) test. This involves extraction of SARS-CoV-2 virus nucleic acid from a specimen, followed by combined reverse transcription of viral RNA and PCR amplification using real-time RT-PCR methods. In silico analysis has demonstrated that Viracor’s SARS-CoV-2 RT-PCR is not expected to crossreact with other coronaviruses. These donor, recipient, and HCW tests include a combination of standard tests (nasal swab, shipped via FedEx and resulted in 12–18 h) and stat tests (nasal swab, transport via same-d courier, resulted in 6 h). We report the type of donor (live donor [LD] or DD), the type of candidate (LD or DD), and type of organ being donated/transplanted.

### Statistical Analysis

All analyses were performed using Stata 16.0/MP for Linux (College Station, TX).

## RESULTS

### Johns Hopkins Health System

Between March 1 and April 15, 2020, 152 ESRD patients in the JHHS underwent diagnostic testing for SARS-CoV-2, among which 31 (20%) were positive (Table 1). The mean age among those who tested positive was 60 ± 15 y. Among ESRD patients who tested positive for SARS-CoV-2, 20 of 31 (65%) were male and 17 of 31 (55%) were African American. Among the 31 ESRD patients who tested positive, 27 of 31 (87%) were alive at the end of the study period. Among KT recipients, 83 had been tested for SARS-CoV-2 and 14 of 83 (17%) were positive; 13 of 14 (93%) with SARS-CoV-2 were alive at the end of the study period.

**TABLE 1.**
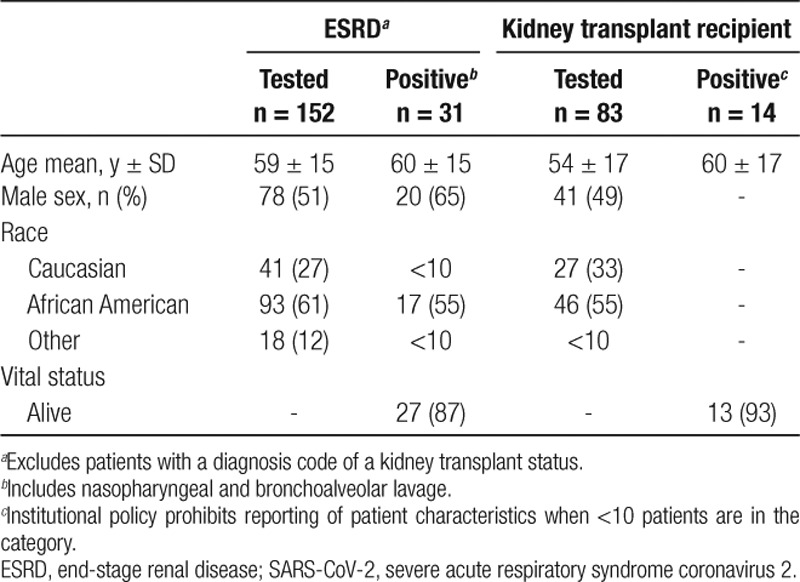
Demographic characteristics and vital status of patients from the Johns Hopkins Health System with ESRD or history of kidney transplant who were tested for SARS-CoV-2 between February 1 and April 14, 2020

### Southwest Transplant Alliance Organ Procurement Organization

Within the donation service area of the STA, between March 1 and April 13, 2020, 92 organs from 27 donors were procured for transplantation (Table 2). All donors tested negative for SARS-CoV-2 through a combination of endotracheal aspirate PCR (25%) and bronchoalveolar lavage (15%). The majority (17 of 27; 63%) of donors were male and Caucasian (15 of 27; 56%); 20 of 27 (74%) were donation after brain death donors. Major causes of death were anoxia (11 of 27; 42%) and head trauma (10 of 27; 38%). Regarding organs transplanted, 42 kidneys, 23 livers, 4 pancreata, 9 hearts, and 14 lungs were transplanted.

**TABLE 2.**
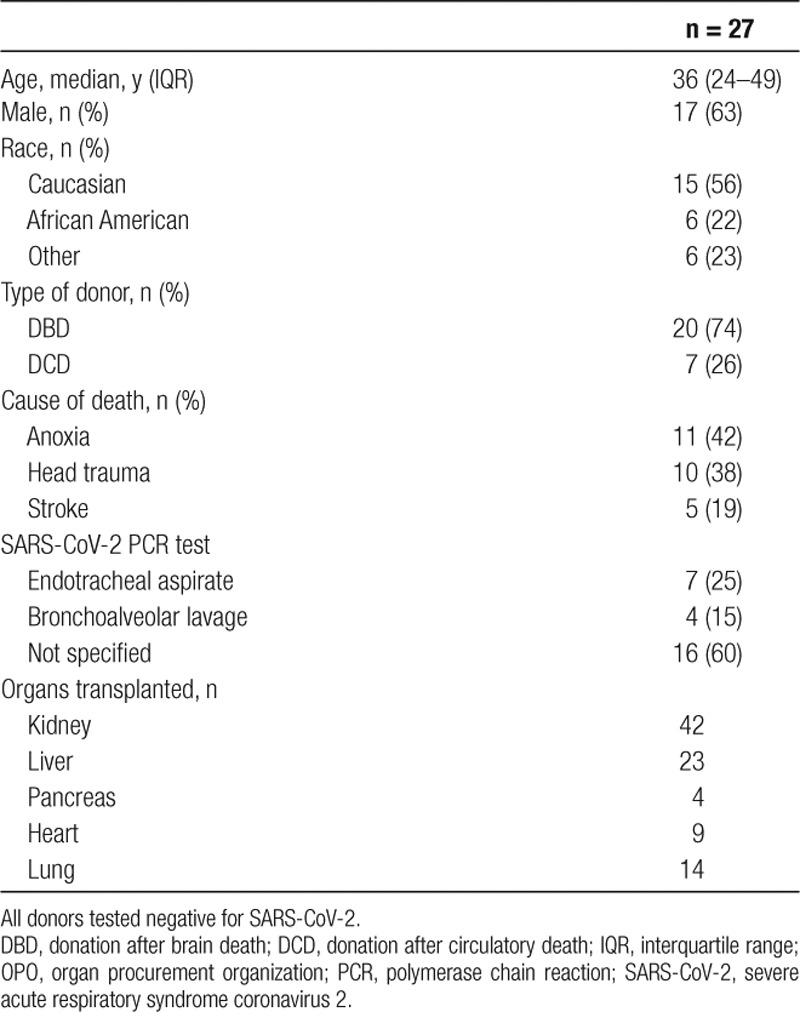
Demographic and clinical characteristics of deceased donors whose organs were procured from Southwest Transplant Alliance OPO donation service area and transplanted between February 23 and April 13, 2020

### NKR and Viracor-Eurofins

All LDs who were screened were negative: 14 kidney donors and 1 liver donor (Table 3). Among 190 potential DDs who were screened, 2 (1%) were positive. Seventeen live kidney donor recipients, 2 LD liver recipients, 13 DD kidney recipients, and 3 kidney-pancreas recipients were screened pretransplant and were all negative. Additionally, 6 HCWs were screened and were negative.

**TABLE 3.**
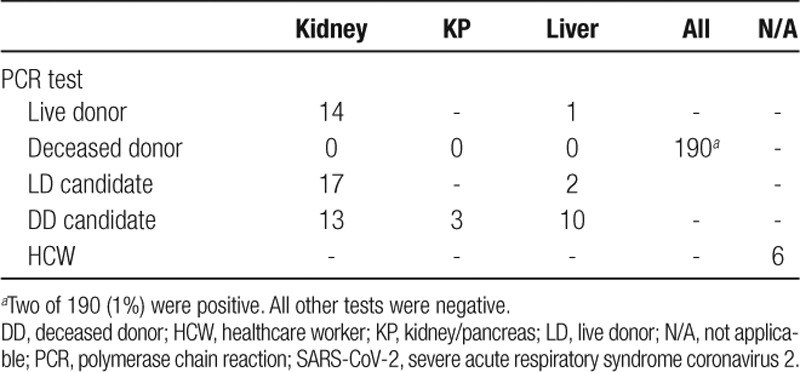
Test characteristics 253 SARS-CoV-2 PCR tests among solid organ transplant donors, recipients, and healthcare workers tested by the National Kidney Registry and Viracor-Eurofins between March 1 and April 15, 2020

## DISCUSSION

In this study of SARS-CoV-2 test results using 3 separate registries, we found low rates of COVID-19 among organ donors and HCWs (0%–1%) who were screened, higher rates of positive diagnostic tests among patients with ESRD or KT (17%–20%), and considerable mortality (7%–13%) among those who tested positive. These findings suggest the threat of COVID-19 for the transplant population is significant and ongoing data collection and reporting is critical to inform transplant practices during and after the pandemic.

COVID-19 is a novel disease and we are learning more about its impact on transplantation in real time. Therefore, early and frequent reporting of incidence and associated mortality is necessary to inform evolving clinical practices. Our findings of low rates of positive screening tests are consistent with the early COVID-19 literature among asymptomatic people who were tested.^23–25^ Furthermore, the higher rates of positive diagnostic tests and mortality among patients with ESRD and KT are also consistent with reports of poorer clinical outcomes among patients with comorbidities.^26–28^ The substantial disparity between rates of SARS-CoV-2 detection among donors and recipients likely reflects that higher-risk patient populations underwent diagnostic tests in greater numbers, compared with those who were screened.

Estimates derived from these datasets, which were not originally designed to answer our research question are, of course, somewhat limited and based on a number of assumptions. First, with regards to electronic medical record from JHHS, the accuracy of coding may be variable. This may have underestimated our results by not accurately capturing ESRD or KT status. Furthermore, early in the pandemic, only symptomatic patients were tested, which may have biased our results by not capturing asymptomatic carriers of the virus. Additionally, as this was a single-health system study, the results may not be generalizable to other populations with different incidence of COVID-19. Second, with regards to the STA data, we were unable to capture transplant recipient COVID-19 data, limiting our inferences about donor-derived SARS-CoV-2. Finally, with regards to the NKR, we only capture SARs-CoV-2 test results. The dataset lacked demographics and pertinent donor information, which limited our ability to investigate incidence further. Additionally, we know there was substantial reductions in living donor transplants at the onset of the pandemic; therefore, this small cohort of living donors and recipients is likely not generalizable to the entire population of living donors in the United States.

In summary, to resume transplantation, it is important to understand the prevalence of COVID-19 among transplant recipients, donors, and HCWs and its associated mortality. Our findings from diverse patient populations demonstrate the risks of COVID-19 in transplantation are significant and stress the importance of ongoing data collection.
